# Finite element analysis of the effect of cementing concepts on implant stability and cement fatigue failure

**DOI:** 10.3109/17453670902947465

**Published:** 2009-06-01

**Authors:** Dennis Janssen, Jantien van Aken, Thierry Scheerlinck, Nico Verdonschot

**Affiliations:** ^1^Radboud University Nijmegen Medical CentreOrthopaedic Research Laboratory, Nijmegenthe Netherlands; ^2^Universitair Ziekenhuis Brussel (UZ Brussel)Department of Orthopaedic Surgery and Traumatology, BrusselsBelgium; ^3^Laboratory for Biomechanical Engineering, University of Twente,EnschedeThe Netherlands

## Abstract

**Background and purpose** Two contradictory cementing techniques (using an undersized stem versus a canal-filling stem) can both lead to excellent survival rates, a phenomenon known as the “French paradox”. Furthermore, previous studies have indicated that the type of bone supporting the cement mantle may affect implant survival. To further evaluate the mechanical consequences of variations in cementing technique, we studied the effect of implant size and type of bone supporting the cement mantle on the mechanical performance of cemented total hip arthroplasty, using finite element analysis.

**Methods** In a generic 2-dimensional plane-strain finite element model of a transverse section of a cemented total hip arthroplasty with a Charnley-Kerboull stem, we varied implant size and type of bone supporting the cement mantle. The models were subjected to 2 × 10^6^ cycles of an alternating loading pattern of torque and a transverse load. During this loading history, we simulated cement fatigue crack formation and tracked rotational stability of the implant.

**Results** Canal-filling stems produced fewer cement cracks and less rotation than undersized stems. Cement mantles surrounded by trabecular bone produced more cement cracks and implant rotation than cement mantles surrounded by cortical bone.

**Interpretation** Our investigation provides a possible explanation for the good clinical results obtained with canal-filling Charnley-Kerboull implants. Our findings also indicate that inferior mechanical properties are obtained with these implants if the cement is supported by trabecular bone, which may be minimized by an optimal cementing technique.

## Introduction

A thin cement mantle (Mann et al. 2004) and cement mantle defects have been associated with the formation of cracks in the cement mantle (Jasty et al. 1991), leading to early failure of total hip arthroplasty (Star et al. 1994). This evidence has resulted in the generally accepted rule of using a stem that is undersized compared to the broach used to prepare the intramedullary canal, to produce a cement mantle that is at least 2 mm thick. Using this technique, excellent survival rates have been obtained (Malchau et al. 2002).

In France in the early 1970s, a surgical technique was developed that contradicted this concept (Langlais et al. 2003, Kerboull et al. 2004). The technique involved the removal of as much trabecular bone as feasible and the implantation of a canal-filling stem in a line-to-line fashion, so that the size of the implant is equal to the size of the broach used to prepare the intramedullary canal. The goal is to transfer loads directly from the stem to the cortical bone, and as such to “protect” the cement mantle (Langlais et al. 2003). The technique results in a very thin cement mantle with multiple defects (Scheerlinck et al. 2006). Surprisingly, this technique also resulted in excellent survival rates (Kerboull et al. 2004, Scheerlinck and Casteleyn 2006). This phenomenon of two seemingly contradictory cementing concepts leading to good outcome has been referred to as the “French paradox” (Langlais et al. 2003).

Although both techniques apparently lead to good clinical results, variations in implant size, cement mantle thickness, and bone type surrounding the cement mantle will cause differences in the response to fatigue loading in terms of implant stability and cement crack formation. Previous studies suggested that large implants may provide superior rotational stability (Massin et al. 2003), and that cement mantles supported by trabecular bone produce inferior results (Ayers and Mann 2003). The aim of our study was to further evaluate the mechanical consequences of variations in cementing technique, using finite element analysis (FEA).

We hypothesized that (1) undersized stems surrounded by a thick intact cement mantle would produce fewer cement fatigue cracks than canal-filling stems, (2) large canal-filling stems would rotate less than undersized stems, and (3) a cement mantle supported by trabecular bone would produce more cement cracks and more implant rotation than a cement mantle supported by cortical bone.

## Material and methods

We created a generic 2-dimensional (2D) plane-strain FEA model of a transverse slice of a Charnley-Kerboull stem replica (CMK; Stratec Medical, Oberdorf, Switzerland) cemented in a cadaver femur. This generic model was subsequently adapted to simulate arthroplasties resulting from various cementing techniques. The FEA models were subjected to a history of fatigue loading, during which crack formation and implant rotation were simulated.

**Figure 1.**
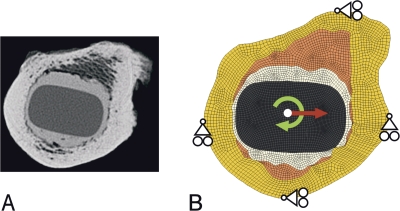
A. The original CT image of a Charnley-Kerboull stem replica cemented line-to-line into a donor femur is shown, which served as the basis for all FEA models. B. An example of an FEA model with a maximal canal-filling stem is shown. From the center of the image to the outer edge, the implant, cement mantle, trabecular bone, and cortical bone are shown. The loading conditions (arrows) and boundary conditions applied during the simulations are also shown.

The model was created from computed tomography (CT) data used previously for geometric analyses of the cement mantle around line-to-line and undersized femoral implants (Scheerlinck et al. 2006). The model was based on a representative example of a Charnley-Kerboull stem implanted in a line-to-line fashion. For the FEA model, an image of the CT data set was taken at the level of the lesser trochanter. In the CT image, the contours of the cortical and trabecular bone, the cement mantle, and the stem were identified as previously described (Scheerlinck et al. 2006). The model was created based on these contours using an automatic mesher (MSC.MARC; MSC Software Corp, Santa Ana, CA). The models had a thickness of 5 mm and consisted of approximately 6,000 8-node brick elements and 12,500 nodal points (Figure 1).

**Figure 2.**
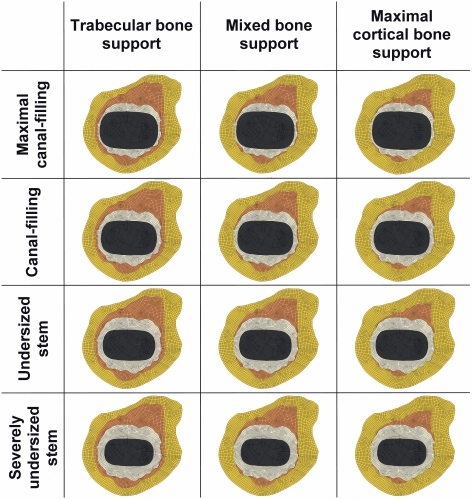
In total, 12 FEA models were created. 4 different stems sizes (resulting in 4 different cement mantles, as shown in the rows) were studied in combination with 3 different bone types supporting the cement mantle (as shown in the columns).

We varied the size of the femoral implant to simulate both canal-filling and undersized implants. The undersized implants were based on the original Charnley-Kerboull implant geometry to exclude variability in the implant design, allowing us to study only the effect of cementing concepts. Considering the cross-sectional geometry of the Charnley-Kerboull stem did not differ much from that of the original Charnley roundback stem, we chose to use scaled-down versions of the original Charnley-Kerboull implant for the models of the undersized stems. Consequently, the cement mantle thickness was varied inversely with femoral component size. 4 cases were created: a model with an incomplete cement mantle (minimum thickness of 0 mm; maximal canal-filling stem), a thin mantle (minimum thickness of 1 mm; canal-filling stem), an average mantle (minimum thickness of 2 mm; undersized stem), and a thick cement mantle (minimum thickness of 3 mm; severely undersized stem) (Figure 2). Due to the typical shape of the implant, the thickness of the cement mantle was minimal in the medial and lateral parts of the reconstruction, while the thickness was greater in the anterior and posterior regions.

The type of bone supporting the cement mantle was varied by changing the material properties of the elements surrounding the cement mantle. 3 variations were analyzed: a cement mantle supported by trabecular bone only (trabecular bone support, representing an implantation technique with poor cement pressurization), a mantle supported by trabecular and cortical bone (mixed bone support, representing an implantation technique with adequate cement pressurization), and a mantle maximally supported by cortical bone (cortical bone support, representing a surgical technique in which most of the trabecular bone is broached away or filled with cement) (Figure 2). To avoid mesh dependency of the results in the simulations, all models were derived from a single generic FEA model. In this model, the mesh architecture was adapted such that all geometric variations in implant size, cement mantle thickness, and type of bone support could be modified by merely changing the material properties assigned to the elements. The material properties of the cortical bone (Lotz et al. 1991), trabecular bone (Kaneko et al. 2004), bone cement (Lewis 1997), and implant were assumed to be isotropic and linear elastic (Table 1). The implant material was modeled with material properties of stainless steel.

**Table 1.** Material characteristics of the different structures of the finite element analysis model

Part of model	Young’s modulus (MPa)	Poisson’s ratio (–)
Stem	210,000	0.3
Bone cement	2,200	0.3
Trabecular bone	1,000	0.3
Cortical bone	7,000	0.4

Contact between the implant and the cement was modeled using a node-to-surface contact algorithm (MSC.MARC). The implant-cement interface was assumed to be debonded from the start of the simulation, implying that no tensile loads could be transferred over the interface, assuming a worst-case scenario. Friction was modeled using a Coulomb stick-slip model with a friction coefficient of 0.25, simulating a satin surface finish for the stem, consistent with the surface finish of the Charnley-Kerboull stems. The cement mantle was assumed to be fixed to the surrounding cortical and trabecular bone.

For 2 × 10^6^ cycles, the models were alternately loaded with a cyclic torque load and a transversal load. The loading configurations were applied in a ratio of 9:1, meaning that during 90% of the loading history a torque load was applied, while during 10% the transversal load was applied. The torque load represented a stair-climbing load, which is critical for implant stability (Bergmann et al. 1995). Since our models were limited to only a slice of an entire reconstruction, the external loads had to be scaled down to the model size. We therefore assumed a torque load of 6.4 Nm acting on the models (Bergmann et al. 1995). The transversal load represented a bending moment in the frontal plane that can be as high as 80 Nm (Bergmann et al. 1995). As a consequence, the implant will exert a medial force on the cement mantle in the proximal region, while more distally lateral forces are transferred to the cement. A transversal load of 400 N acting in the medial direction represented bending in the frontal plane in our 2D models. Displacement in the anteroposterior direction was restricted in the medial and lateral part of the outer cortex, while displacement in the mediolateral direction was restricted in the anterior and posterior part of the outer cortex (Figure 1B). In this manner, deformation and expansion of the cortical bone was allowed, enabling movement and deformation of the stem, cement, and bone, while rigid body displacement of the models was restricted.

Because only a slice of an entire reconstruction was analyzed, a plane-strain state was assumed in the model. Although 2D elements are usually used in such a case, we used 3-dimensional (3D) brick elements to make the FEA models compatible with our fatigue crack formation algorithm. To compensate for this, all nodes on the top and bottom planes of the model were fixed in the axial direction.

Fatigue crack formation and creep were simulated using a custom-written algorithm based on FEA (Stolk et al. 2004). Based on the local cement stress situation and the number of loading cycles, a small crack could occur at a certain location in the mantle. This crack was then accounted for mechanically by locally reducing the stiffness to virtually zero in the direction perpendicular to the crack. At the same location, an additional second and third crack could be formed, perpendicular to the first crack. Furthermore, during the simulation small cracks could propagate, thereby forming macrocracks that could eventually span the full thickness of the cement mantle. Similarly, creep deformation was simulated to occur locally in the cement mantle, also based on the local cement stress and the number of loading cycles. The formation of bone cement cracks was determined using so-called S-N curves (Murphy and Prendergast 1999, 2002), whereas the amount of local creep strain in the cement mantle was calculated using a creep law (Verdonschot and Huiskes 1995). This creep-damage algorithm has been used previously to differentiate between the survival of various implant designs (Janssen et al. 2005, Stolk et al. 2007).

During the simulations, we monitored the number of cracks formed in the cement mantle. In order to enable comparisons between the various models, the number of cracks was normalized by dividing by the number of cracks that would ultimately be possible in the cement. The total number of cement cracks possible in the cement mantle depended on the size of the implant, and ranged from 17,500 to 38,000 for the models with the largest and smallest implants, respectively.

In addition, the rotation of the femoral component with respect to the cortical bone was calculated and was considered a measure of the level of implant stability. To calculate implant rotation, initial elastic deformations of the models were ignored—to display only the long-term effect of creep and crack formation on implant rotation. To demonstrate the effect of type of bone supporting the cement mantle, formation of cement damage and implant rotation as predicted by models with trabecular and cortical bone support were calculated and presented relative to the results of models with mixed bone support, which was considered to be the standard situation.

## Results

**Figure 3.**
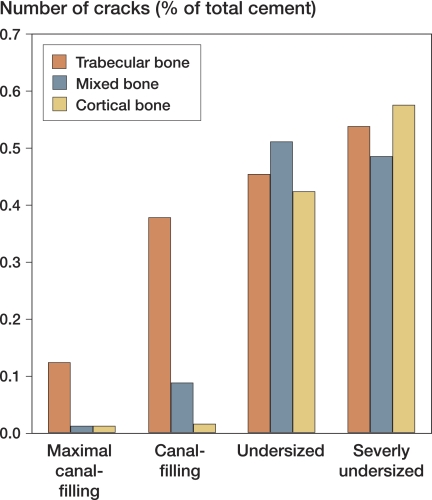
In contrast to our first hypothesis, after 1.25 × 10^6^ loading cycles, the models with undersized stems produced more cement cracks than models in which a canal-filling stem was simulated. The number of cracks was normalized by dividing by the maximal number of cracks that could possibly be simulated in the cement mantle.

**Figure 4.**
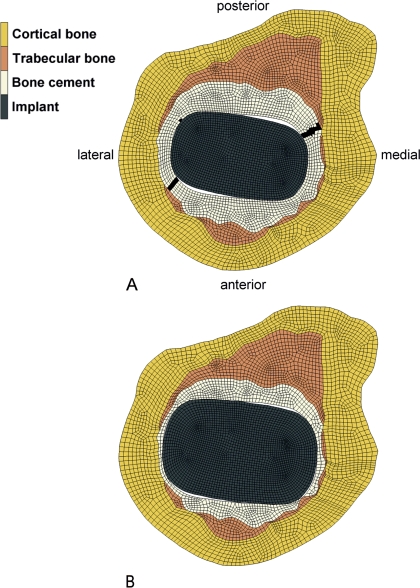
In contrast to our first hypothesis, the models with an undersized stem produced more cracks in the cement mantle, leading to large cracks in the posteromedial and anterolateral corners of the cement mantles. The cracks are shown in the figure as black areas in the cement mantle. In (A), an FEA model with an undersized stem and mixed bone support is shown with two full-thickness cracks in the cement mantle, whereas in (B), which depicts an FEA model with a maximal canal-filling stem and mixed bone support, almost no cracks were formed during the simulation. The deformations have been magnified by a factor of 10 for illustrative purposes.

In contrast with our first hypothesis, the canal-filling stems produced fewer cracks in the cement mantle than the undersized stems (Figure 3). In general, the number of cracks formed in the cement mantle increased with decreasing size of the implant. Cyclic torque loading of the models caused cracks to appear in the cement mantle at the posteromedial and anterolateral corners of the stem (Figure 4). Cracks that crossed the full thickness of the cement mantle appeared first in the anterolateral corner of the cement mantle, which was followed in some cases by a secondary crack in the posteromedial corner. We observed full-thickness cracks in all models with undersized implants, whereas in the models with the maximal canal-filling implant, full-thickness cracks occurred only when the cement mantle was supported by trabecular bone. In two models with a severely undersized stem (cement mantle supported by mixed bone and cortical bone), full-thickness cracks prevented the model from converging after 1.25 × 10^6^ cycles. Deformations in these models, in combination with the alternating loading profile, caused instabilities in the contact algorithm at the implant-cement interface. Differences in crack formation and implant rotation between models were therefore investigated at 1.25 × 10^6^ cycles instead of at 2 × 10^6^ cycles.

**Figure 5.**
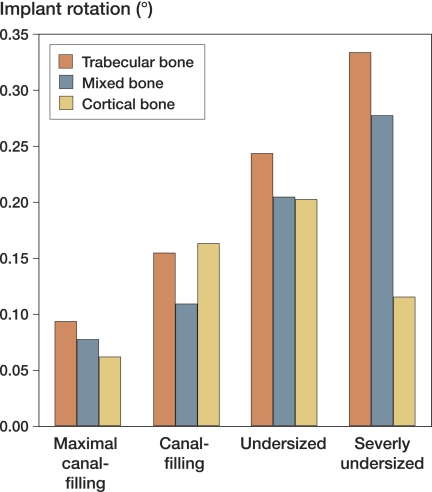
Consistent with our second hypothesis, after 1.25 × 10^6^ loading cycles the maximal canal-filling stem rotated less than the undersized stem. The rotation values represent the rotation resulting from creep and crack formation in the cement mantles, since elastic deformations were omitted.

Consistent with our second hypothesis, after 1.25 × 10^6^ loading cycles, the canal-filling stems had rotated less than the undersized stems (Figure 5). Creep and crack formation in the cement mantle caused progressive rotation of the stem, particularly during the first 1 × 10^6^ cycles. In some models, sudden increases in implant rotation occurred when changing from the torque load to the transversal load. When the transversal load was subsequently reapplied, the stem settled again in a new and more stable position, and implant rotation decreased again.

In general, models with a cement mantle supported by trabecular bone produced more cracks in the cement mantle and caused more implant rotation than the models in which the cement mantle was supported by a mixture of trabecular and cortical bone. In addition, increasing cortical bone reduced implant rotation and reduced the number of cement cracks (Table 2).

**Table 2.** Relative effect of type of bone support for the cement mantle on cement damage and implant rotation, compared to a situation in which the cement mantle is supported by mixed bone

	Trabecular bone support	Cortical bone support
Cement damage	+331%	–20%
Implant rotation	+25%	–6%

## Discussion

Although the FEA model we used was based on accurate and clinically relevant data for the Charnley-Kerboull stem, it obviously had certain limitations. In our study, we used a 2D model rather than a 3D one—to limit the computational costs while providing sufficient detail for analysis of the effects of changes in the cement mantle geometry. This limited the loads we could apply to in-plane loads, such as a torque load. The effect of axial loads, leading to implant subsidence and tangential stresses in the cement mantle, was not simulated in our models. However, it has been demonstrated that torque resulting from stair-climbing activities is the most detrimental load for cement mantle failure (Bergmann et al. 1995). Moreover, implant subsidence may have been limited for the implant design we analyzed, considering it has a collar.

Our model was based on a single cross section at the level of the lesser trochanter, not taking into account differences in the cross-sectional shape of other parts of the implants. However, our findings are similar to those of an FEA investigation of Massin et al. (2003) who used 3D FEA models of entire cemented reconstructions to analyze the effects of implant-cement bond and implant size. In that study, the proximal canal fill of implants was varied (100% to 90% to 80% to 70% of the optimal fill). The results of that study showed that an optimal fill (large implant) increased the rotational stability. In addition, they demonstrated that loads are mainly transferred in the proximal region of the reconstruction, which provides further justification for our choice of performing analyses at the level of the lesser trochanter. Unfortunately, to our knowledge no data are available on experimental mechanical testing or implant retrieval analysis against which we can verify our findings.

Regardless of the fact that only one level of the cemented reconstruction was analyzed, our results may to some extent have been dependent on the specific geometry that we used. We modeled a specific cross section of the CT dataset rather than creating an average shape, because we expected that a specific geometry would enable our models to differentiate better between the various cases. We selected a “representative” cross section from a previous study (Scheerlinck et al. 2006). This cross section comprised typical features of line-to-line reconstructions, such as a thin cement mantle in the antero-medial region (Scheerlinck et al. 2006).

An additional limitation to our study was the fact that we only analyzed the Charnley-Kerboull implant, even though it is used most widely when performing line-to-line reconstructions (Scheerlinck and Casteleyn 2006). Consequently, our results and subsequent conclusions only apply to this implant. This choice limited the scope of our work, since we did not analyze variations in design such as implant shape and surface roughness. Such variations may have consequences for the implant-cement bond, implant subsidence, and cement mantle abrasion. In our study, however, we assumed that the stem was not bonded to the cement mantle from the start of the simulation, as several studies have shown that implant-cement debonding occurs relatively early in the lifespan of a cemented reconstruction (Jasty et al. 1991). In addition, variations in the surface roughness of an implant may affect cement mantle abrasion. For instance, polished, collarless implants may be more susceptible to subsidence and micromotions than collared implants with a high degree of surface roughness, although they may produce less abrasive wear debris (Verdonschot and Huiskes 1998). These phenomena were not included in the current calculations.

In the models with an undersized stem and maximal cortical cement mantle support, we assumed that all trabecular bone was filled with bone cement. As a result of the lack of fatigue data on interdigitated cement, this interdigitated region was represented in the FEA model by material properties of bone cement, although its strength may be lower than that of pure bone cement (Race et al. 2003). Thus, our FEA model possibly over-predicted the mechanical properties of the cement surrounding the undersized stems in the case of maximal cortical bone support.

Based on the excellent survival rates (Malchau et al. 2002), one would expect that a thick, intact cement mantle would be more advantageous than a cement mantle with defects. In contrast, our data indicate that a canal-filling stem performs better than an undersized implant. This may be explained by the fact that when using a larger implant, the loads applied to the implant are transferred over a larger stem-cement interface, reducing cement stresses and fatigue crack formation. In addition, direct load transfer from implant to femoral bone may reduce the cement stresses further. This suggests that decreasing the stem size to achieve a thicker cement mantle may not always pay, at least not from a mechanical point of view. As such, our results give a possible explanation for the good results obtained by surgeons adhering to the line-to-line implantation technique.

Biological factors such as postoperative bone remodeling and particle-induced osteolysis were not taken into account in our simulations. For example, periprosthetic bone resorption may be more pronounced in reconstructions with canal-filling implants, thereby affecting the mechanical behavior. Furthermore, the larger number of cement mantle defects around canal-filling stems (Scheerlinck et al. 2006) may be detrimental in vivo, because they allow easier access of cement and polyethylene debris particles to the bone-cement interface, inducing osteolysis (Maloney et al. 1990). Hence, from this perspective, undersized stems would be beneficial. On the other hand, undersized stems caused full-thickness cement mantle cracks to occur earlier, thereby also creating early pathways for particles to reach the surrounding bone. Nonetheless, biological processes that play a role in vivo may provide an additional explanation for why undersized stems are so successful, while in this study they were inferior to canal-filling stems.

Our data indicate that trabecular bone support results in a mechanically inferior cement mantle. These data are consistent with those of Ayers and Mann (2003), who reported that trabecular bone support elevates the stresses in the cement mantle. This emphasizes the importance of the use of pressure lavage and adequate cement pressurization in order to achieve maximal cement penetration into cancellous bone, if possible up to the stiff inner cortex.

In conclusion, our data suggest that (1) undersized stems surrounded by a thick, intact cement mantle produce more cement fatigue cracks than canal-filling stems surrounded by a thin cement mantle, (2) large canal-filling stems rotate less than undersized stems, and (3) a cement mantle supported by trabecular bone produces more cement cracks and implant rotation than a cement mantle supported by cortical bone.
